# Pilot Study on the Role of Circulating miRNAs for the Improvement of the Predictive Ability of the 2MACE Score in Patients with Atrial Fibrillation

**DOI:** 10.3390/jcm9113645

**Published:** 2020-11-12

**Authors:** José Miguel Rivera-Caravaca, Raúl Teruel-Montoya, Vanessa Roldán, Rosa Cifuentes-Riquelme, José Antonio Crespo-Matas, Ascensión María de los Reyes-García, Sonia Águila, María Piedad Fernández-Pérez, Laura Reguilón-Gallego, Laura Zapata-Martínez, Nuria García-Barberá, Vicente Vicente, Francisco Marín, Constantino Martínez, Rocío González-Conejero

**Affiliations:** 1 Department of Cardiology, Hospital Clínico Universitario Virgen de la Arrixaca, University of Murcia, Instituto Murciano de Investigación Biosanitaria (IMIB-Arrixaca), CIBERCV, 30120 Murcia, Spain; jmrivera429@gmail.com (J.M.R.-C.); fcomarino@hotmail.com (F.M.); 2 Department of Hematology and Medical Oncology, Hospital General Universitario Morales Meseguer, University of Murcia, Centro Regional de Hemodonación, Instituto Murciano de Investigación Biosanitaria (IMIB-Arrixaca), 30003 Murcia, Spain; raulteruelmontoya@hotmail.com (R.T.-M.); vroldans@gmail.com (V.R.); rcifuentesriquelme@gmail.com (R.C.-R.); jocresma@hotmail.com (J.A.C.-M.); sregapa@gmail.com (A.M.d.l.R.-G.); sonia.aguila@um.es (S.Á.); mpfernandezperez@gmail.com (M.P.F.-P.); reguilongallegolaura@gmail.com (L.R.-G.); laurazap97@gmail.com (L.Z.-M.); nurgarbar@gmail.com (N.G.-B.); vicente.vicente@carm.es (V.V.); 3 CIBERER (U765), 30003 Murcia, Spain

**Keywords:** miRNAs, atrial fibrillation, risk stratification, adverse cardiovascular event, 2MACE

## Abstract

*Background*. Atrial fibrillation (AF) increases the risk for stroke but also for non-stroke major adverse cardiovascular events (MACE). The 2MACE score was recently proposed to predict these events. Since the interest of microRNAs (miRNAs) in cardiovascular diseases is increasing, we aimed to investigate whether miRNA levels may improve the predictive performance of the 2MACE score. *Methods*. We included consecutive AF patients stable on vitamin K antagonist therapy. Blood samples were drawn at baseline and plasma expression of miRNAs was assessed. During a median of 7.6 (interquartile range (IQR) 5.4–8.0) years, the occurrence of any MACE (nonfatal myocardial infarction/cardiac revascularization and cardiovascular death) was recorded. *Results*. We conducted a miRNA expression analysis in plasma from 19 patients with and without cardiovascular events. The miRNAs selected (miR-22-3p, miR-107, and miR-146a-5p) were later measured in 166 patients (47% male, median age 77 (IQR 70–81) years) and all were associated with a higher risk of MACE. The addition of miR-107 and miR-146a-5p to the 2MACE score significantly increased the predictive performance (c-indexes: 0.759 vs. 0.694, *p* = 0.004), and the model with three miRNAs also improved the predictive performance compared to the original score (c-indexes: 0.762 vs. 0.694, *p* = 0.012). 2MACE models with the addition of miRNAs presented higher net benefit and potential clinical usefulness. *Conclusions*. Higher miR-22-3p andmiR-107 and lower miR-146a-5p levels were associated with a higher risk of MACE. The addition of these miRNAs to the 2MACE score significantly increased the predictive performance for MACE, which may aid to some extent in the decision-making process about risk stratification in AF.

## 1. Introduction

Atrial fibrillation (AF) is the most frequent cardiac arrhythmia and it implies a high morbidity and mortality [1,2]. There is evidence supporting the presence of a prothrombotic or hypercoagulable state in AF, which is associated with an increased risk of stroke and thromboembolism [3]. However, a non-negligible proportion of patients suffer from adverse cardiovascular events regardless of stroke, including myocardial infarction (MI) [4]. For this reason, the 2MACE score was proposed to predict major adverse cardiovascular events (MACE; MI, cardiac revascularization, and cardiovascular death) in AF patients [5], and external validations have shown promising results [6,7].

On the other hand, the study of biomarkers as prognostic or risk stratification markers has gained attention. In this context, microRNAs (miRNAs) are post-transcriptional regulators that have been widely recognized as active effectors of the cardiovascular system [8]. The recognition of miRNAs as plasma biomarkers of cardiovascular disease is being consolidated because their collect is minimally invasive, only a blood sample is required, and they are remarkably stable in the bloodstream, and sensitive to acute or chronic environmental alterations [9,10]. In addition to their great accessibility, circulating miRNAs are relatively easy to measure [11]. While a few works have reported profiles of circulating miRNAs as predictors for cardiovascular events in patients presenting MI [12], coronary artery disease [13], or acute coronary syndrome [14], their role as prognostic markers in AF is still unknown.

In the present study, we aimed to determine a profile of circulating miRNAs with the ability to predict MACE in AF, and to assess whether plasma miRNA levels can improve the predictive performance of the 2MACE score in a “real-world” cohort of AF patients under vitamin K antagonist (VKA) therapy.

## 2. Methods

We included a prospective cohort of permanent/paroxysmal AF patients from our outpatient anticoagulation clinic in a tertiary hospital (Murcia, southeast of Spain). All patients were on VKA therapy with stable international normalized ratios (INR 2.0–3.0) during at least the previous 6 months. This 6-month period of good anticoagulation control with VKA before inclusion was required to have a baseline homogeneity that would allow us to avoid the potential bias of poor anticoagulation control on clinical outcomes. Patients with prosthetic heart valves, rheumatic AF, or patients with acute coronary syndrome, stroke (ischemic or embolic), unstable chest pain, hemodynamic instability, hospital admission, or surgical intervention in the preceding 6 months were excluded.

The recruitment period was from May 2007 to December 2007. At inclusion, a complete medical history was recorded, and stroke risk (CHA_2_DS_2_-VASc) and bleeding risk (HAS-BLED) were estimated. The time in the therapeutic range at 6 months after entry was calculated using the Rosendaal method.

All subjects who met eligibility criteria were enrolled after providing written informed consent. The study was approved by the ethical committee of our institution (code: AVAL03/11) and performed in accordance with the ethical standards laid down in the 1964 Declaration of Helsinki and its subsequent amendments.

### 2.1. Assessment of the 2MACE Score

The baseline 2MACE score was retrospectively calculated in all patients as described by Pastori et al. [5]. This score was described as a simple risk score to identify AF patients with a high residual risk of cardiovascular events and to improve cardiovascular risk stratification. The 2MACE score includes 2 points for metabolic syndrome and age ≥75, and 1 point for MI/revascularization, congestive heart failure (ejection fraction ≤40%) and thromboembolism (stroke/transient ischemic attack). The original publication stated that the 2MACE score was proposed to predict MACE (i.e., any of the following: MI, cardiac revascularization, and cardiovascular death) in AF patients.

### 2.2. Blood Samples Collection and miRNome Analysis

Blood samples were atraumatically drawn at study entry and without stasis into syringes preloaded with trisodium citrate (0.011 mol/L), i.e., all patients were stable under VKA therapy for at least 6 months when the blood sample was collected. Samples were centrifuged at 2200× *g* and 4 °C for 10 min, and the supernatants were stored in aliquots at −80 °C until further use. miRNAs from plasma were purified using the Nucleo Spin miRNA Plasma Kit (Macherey–Nagel) in accordance with the manufacturer’s protocol. Plasma miRNAs were screened using the plasma/serum focus microRNA PCR Panel V4 (Exiqon) composed of 179 miRNAs as has been previously described [15]. Data were normalized by cycle threshold (Ct) mean value for miR-103a and miR-191-5p, which showed a high correlation (Pearson correlation) with both the global geometrical mean value and the top 20 expressed miRNA geometric mean values, resulting in an r = 0.8607 (*p*-value < 0.0001) and r = 0.8902 (*p*-value < 0.0001), respectively. Plasma expression of miRNAs was calculated using the 2^−ΔCt^ method.

### 2.3. Follow-Up and Endpoints

The primary endpoint was the occurrence of any MACE (the composite of nonfatal MI or cardiac revascularization and cardiovascular death (death caused by sudden death, progressive congestive heart failure, fatal MI, or procedure-related death), during the follow-up period. We excluded from MACE all embolic events; i.e., stroke, transient ischemic attack, and peripheral or systemic embolism.

The median follow-up was 7.6 (interquartile range (IQR) 5.4–8.0) years. Follow-up was performed through routine visits to the anticoagulation clinic, the hospital electronic medical records, or, when unavailable, by telephone interview. No specific visits were performed regarding the study. Of note, no patient was lost to follow-up. The investigators identified, confirmed, and recorded all adverse events and outcomes.

### 2.4. Statistical Analysis

Continuous variables were presented as mean±SD or median (interquartile range (IQR)), according to the Kolmogorov–Smirnov test. Categorical variables were presented as absolute frequencies and percentages. The Pearson χ2 test was used to compare frequencies and comparisons of miRNA levels were analyzed using the Mann–Whitney *U* test or the Student *t*-test, as appropriate.

Cox regression analyses were performed to investigate the association of each miRNA with the risk of MACE.

Receiver operating characteristic curves were used to evaluate the predictive ability (expressed as c-indexes) of the original 2MACE score and the miRNA-modified ones. The cut-off point of each miRNA with the best combination of specificity and sensitivity was assessed by the Youden index. Comparisons of receiver operating characteristic curves were performed as described by DeLong et al. [16].

Discrimination and reclassification performances were evaluated by calculating the integrated discrimination improvement (IDI) and the net reclassification improvement (NRI), according to the methods of Pencina et al. [17].

The clinical usefulness and the net benefit of the original score in comparison with the miRNA-modified scores was estimated by using the decision curve analysis (DCA), as was proposed by Vickers et al. [18]. The DCA shows the clinical usefulness of each new model based on a continuum of potential thresholds for adverse events (*x* axis) and the net benefit of using the model to stratify patients at risk (*y* axis) relative to assuming that no patient will have an adverse event. Here, those models that are the farthest away from the slanted dashed line (i.e., assumes all MACEs) and the horizontal line (i.e., assumes no MACE) at a particular threshold probability demonstrate the higher net clinical benefit.

A *p*-value < 0.05 was accepted as statistically significant. Statistical analyses were performed using SPSS 21.0 (SPSS Inc., Chicago, IL, USA), MedCalc v. 16.4.3 (MedCalc Software bvba, Ostend, Belgium), STATA v. 12.0 (Stata Corp, College Station, TX, USA), and survIDINRI package for R v. 3.3.1 for Windows.

## 3. Results

### 3.1. Pilot Study

**Figure 1:**
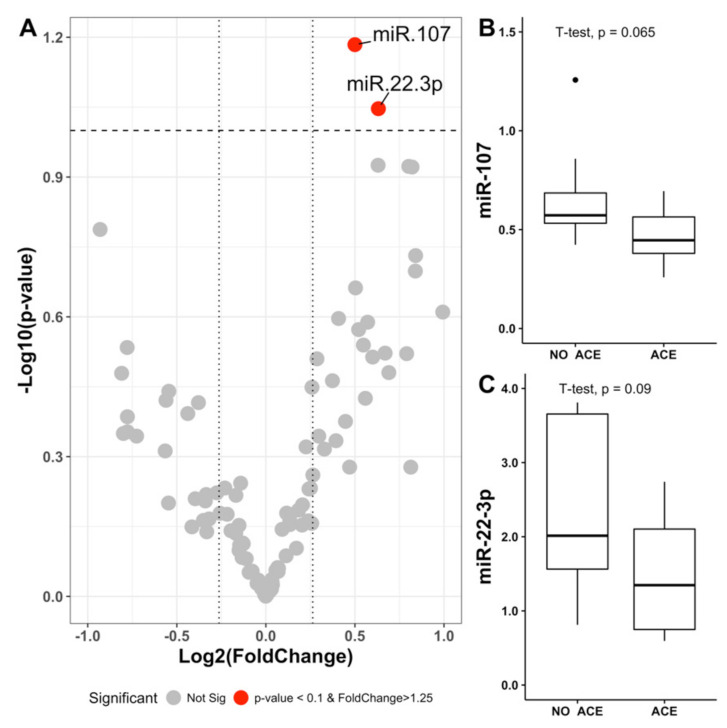
Levels and selection of miRNAs in the pilot study. (**A**) Volcano plot representing the fold change values in logarithm of base 2 (X-axis) and the *p*-values in logarithm of base 10 (Y-axis). (**B**,**C**) Box plots representing the miRNA levels selected for meeting the criteria of *p*-value less than 0.1 and fold change greater than 1.25. ACE = adverse cardiovascular events.

To test if miRNA levels were associated with the development of cardiovascular events, we firstly conducted a miRNA expression pattern analysis in plasma from 9 patients with and 10 patients without cardiovascular events. Since ischemic stroke is the classic main efficacy outcome in AF, we initially selected this endpoint to homogenize the samples. As shown in Supplementary Table S1, cases with stroke and controls without stroke were matched and no clinical differences between these two groups were observed. Of the 178 miRNAs on the panel, we selected those that were detected in all the samples (n = 110) for further analysis. Assuming the criteria of: (i) Fold change in log2 greater than 1.25 and (ii) a statistical significance level of 0.1, due to the limited number of samples; differences of plasma miRNA levels between cases and controls were found only for miR-22-3p and miR-107 (Figure 1). In addition to the two miRNAs selected from this pilot study, we also included miR-146a-5p in the validation study due to its role in the development of cardiovascular events in AF patients [19,20].

### 3.2. Validation Study

The three miRNAs selected were measured in 166 patients (47% male, median age 77 (IQR 70–81) years). The median CHA_2_DS_2_-VASc and HAS-BLED were 4 (IQR 3–5) and 2 (IQR 2–3), respectively, whereas the median 2MACE score was 2.5 (IQR 1–4). A summary of baseline clinical characteristics is shown in Table 1.

We first aimed to find an association between the levels of these three miRNAs and MACE in the cohort of 166 patients. During a median of 7.6 (IQR 5.4–8.0) years, 49 (29.5%; annual rate 3.88%/year) patients suffered a MACE. As shown in Table 2, all miRNAs were associated with the risk of MACE. Hence, miR-22-3p presented a hazard ratio (HR) of 1.07 (95% CI 1.02–1.14), miR-107 presented an HR of 3.66 (95% CI 1.19–11.24), and miR-146a-5p showed an inverse association with MACE, with an HR of 0.86 (95% CI 0.74–0.99).

When the predictive ability for MACE was tested, receiver operating characteristic (ROC)curve analyses demonstrated that miR-22-3p and miR-107 presented poor c-indexes (0.523; 95% CI 0.431–0.632 and 0.555; 95% CI 0.476–0.632, respectively) whereas miR-146a-5p exhibited a moderate c-index (0.656; 95% CI 0.578–0.728). In order to test if any of these miRNAs could enhance the predictive ability of the 2MACE score, we identified cut-off points with the best combination of sensitivity and specificity. The best cut-offs were 0.191, 0.137, and 1.979 for miR-22-3p, miR-107, and miR-146a-5p, respectively. Therefore, patients with miR-22-3p or miR-107 levels over the cut-off points and patients with miR-146a-5p levels under the cut-off point were categorized as being at “high risk” of MACE.

**Figure 2:**
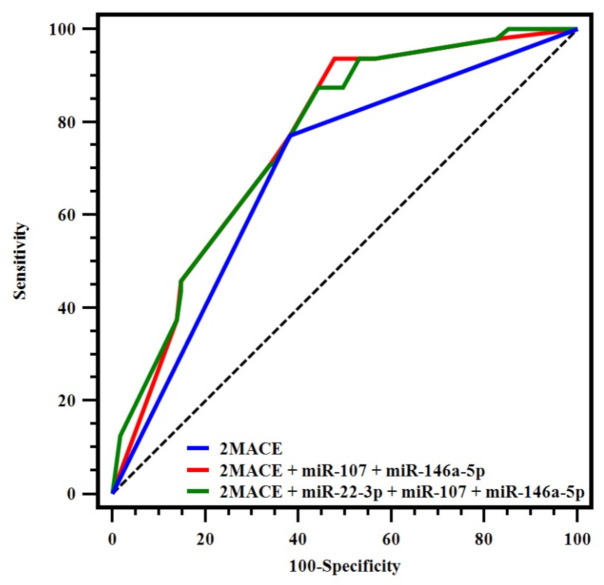
Receiver operating characteristic-curves for the 2MACE score and the 2MACE models with the addition of miRNAs.

Combinations of miRNAs were then included in the 2MACE score, according to the previously established miRNA risk category. Thus, the addition of miR-107 and miR-146a-5p to the 2MACE significantly increased the predictive performance (c-indexes: 0.759 vs. 0.694, *p* = 0.004). Although miR-22-3p alone showed a poor c-index, we also tested a model with this miRNA in addition to miR-107 and miR-146a-5p into the 2MACE score. Similarly, this model also improved the predictive performance of the original 2MACE (0.762 vs. 0.694, *p* = 0.012). In both models of miRNAs, the sensitivity was also enhanced, as is shown by the results of IDI (Table 3, Figure 2).

**Figure 3:**
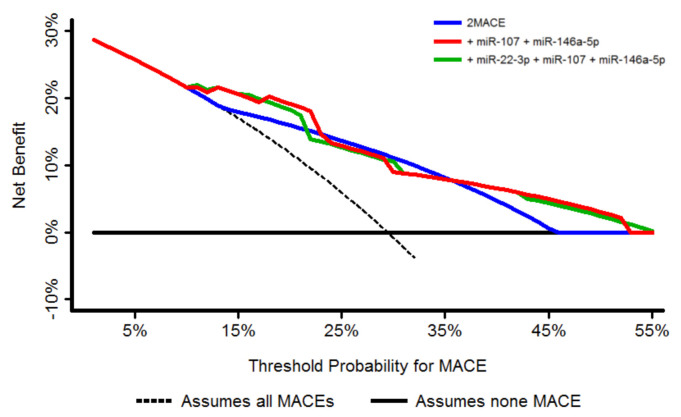
Decision curve analysis for the 2MACE score and the 2MACE models with the addition of miRNAs. MACE = major adverse cardiovascular event.

Finally, a DCA was plotted, showing that both 2MACE models with the addition of miRNAs presented higher net benefit and clinical usefulness compared to the original 2MACE for a large threshold of probabilities (from 10 to 25% and 35 to 55%) (Figure 3).

## 4. Discussion

In this study including steadily anticoagulated AF patients taking VKAs, we observed that plasma levels of miR-22-3p, miR-107, and miR-146a-5p were associated with a higher risk of MACE. Importantly, the predictive ability of the 2MACE score for this event was enhanced with the addition of miRNAs.

During the last years, the interest of miRNAs in AF is increasing [21,22,23]. Despite most of the evidence suggesting a relationship of miRNAs with incident AF or recurrent AF after ablation [24,25,26,27], the therapeutic potential of miRNAs in AF has also emerged [28,29].

For example, miR-22-3p showed to play a role in several cardiovascular diseases [30]. Thus, a recent study has demonstrated that miR-22-3p plasma levels tend to be higher in patients with AF and a high CHA_2_DS_2_-VASc score. This suggests that this miRNA is more expressed in patients with high thromboembolic risk, which could provide some information about the pathophysiological conditions of AF patients [31]. Previously, elevated miR-22-3p serum levels have been shown in patients with heart failure, suggesting that high miR-22-3p circulating levels in serum may reflect intracellular levels in certain tissues and thus may explain the potential functional effect of the miRNA [32]. This is of particular importance since different in vivo models have shown that miR-22-3p overexpression promotes heart hypertrophy while downregulation protects mice from the pathology [33,34]. It is thus tempting to speculate that the higher levels of miR-22-3p in patients with MACE may induce a functional effect in tissues, still to be determined, and therefore may not act only as a mere biomarker of comorbidities.

In another recent study, miR-107 was investigated in AF patients undergoing catheter ablation. Although this miRNA was downregulated in sera from patients with AF recurrence compared to patients without AF recurrence, differences in expression were not significant [35]. However, the role of miR-107 in cardiovascular diseases is not well documented.

There is, however, more prognosis information regarding miR-146a-5p. A preliminary analysis of patients from this cohort at ~3 years of follow-up already showed an association of miR-146a-5p with adverse cardiovascular events (any of the following: Stroke/transient ischemic attack, systemic embolism, MI, and acute heart failure). In that study, lower levels of miR-146a-5p due to the presence of a functional single-nucleotide polymorphism rs2431697 were related to poor prognosis [20]. These data have been later confirmed at ~8 years, where miR-146a-5p was suggested as a regulator of neutrophil extracellular trap formation, and thus associated with cardiovascular risk [19]. Then, it could be hypothesized that dysregulation of miRNAs involved in inflammation may be associated with a higher risk of adverse events in patients with AF [36,37].

Nevertheless, the clinical usefulness of biomarkers when they are evaluated alone may be only limited. In fact, a common criticism of biomarkers is their high variability and lack of specificity in different clinical settings [38,39,40]. However, an advantage of miRNAs compared to other biomarkers is their plasma stability, which makes them less likely to be altered due to acute conditions [9,41], even though results from miRNAs levels should be interpreted with caution and contextualized according to clinical risk factors. In the present analysis, we considered this approach and included several miRNAs into the 2MACE score, which is a clinical score encompassing traditional risk factors for MI. In a similar way, a previous study also suggested that the use of a scoring system that would incorporate both circulating biomarkers and clinical factors might be more useful [42].

### Limitations

There are some limitations that we aim to acknowledge. First, this study was performed in a single center, and it was composed of a Caucasian-based population. Second, all patients were hemodynamically stable and had good anticoagulation control during the previous 6 months after entry, in an attempt to homogenize the sample and avoid the potential impact of acute conditions or poor anticoagulation control in the development of adverse events. Therefore, our results might not be extrapolated to different clinically contexts. In addition, our study is limited only to AF patients treated with VKAs, since all patients were under this therapy at inclusion. Although VKAs are still the most commonly prescribed oral anticoagulants in Spain, the use of direct-acting oral anticoagulants (DOACs) is increasing worldwide. Thus, our results require further validation in AF patients under VKAs and DOACs in order to broaden their clinical usefulness. Finally, we only measured miRNAs at inclusion, and no other determinations have been performed throughout the follow-up. Comorbidities change over time and how miRNAs could evolve during the follow-up period has not been determined. For the above reason, further studies are warranted to confirm our results.

## 5. Conclusions

In this study including AF patients taking VKAs, we found that higher miR-22-3p and miR-107 and lower miR-146a-5p plasma levels were associated with higher risk of MACE at follow-up. The addition of these miRNAs to the 2MACE score significantly increased the predictive performance for MACE and enhanced its clinical usefulness. A combination of miRNAs and clinical risk factors could aid to some extent in the decision-making process about risk stratification in patients with AF.

## Figures and Tables

**Table 1:** Validation study cohort baseline clinical characteristics.

	Overall N = 166	Patients Without MACE N = 117	Patients with MACE N = 49	*p*
Demographic				
Male sex, n (%)	78 (47.0)	54 (46.2)	24 (49.0)	0.739
Age (years), median (IQR)	77 (70–81)	74 (68–79)	80 (77–84)	<0.001
Comorbidities, n (%)				
Hypertension	140 (84.3)	96 (82.1)	44 (89.8)	0.210
Diabetes mellitus	41 (24.7)	25 (21.4)	16 (32.7)	0.124
Heart failure	68 (41.0)	37 (31.6)	31 (63.3)	0.001
History of stroke/TIA/thromboembolism	32 (19.3)	17 (14.5)	15 (30.6)	0.016
Renal impairment	13 (7.8)	6 (5.1)	7 (14.3)	0.045
Coronary artery disease	36 (21.7)	22 (18.8)	14 (28.6)	0.163
Hypercholesterolemia	54 (32.5)	41 (35.0)	13 (26.5)	0.286
Current smoking habit	26 (15.7)	12 (10.3)	14 (28.6)	<0.01
Current alcohol consumption	3 (1.8)	3 (2.6)	0 (0.0)	0.622
History of previous bleeding	12 (7.2)	5 (4.3)	7 (14.3)	0.052
Concomitant treatment, n (%)				
Amiodarone	13 (7.8)	10 (8.5)	3 (6.1)	0.596
Digoxin	28 (16.9)	17 (14.5)	11 (22.4)	0.214
Calcium antagonist	41 (24.7)	24 (20.5)	17 (34.7)	0.053
Beta-blockers	53 (31.9)	39 (33.3)	14 (28.6)	0.548
Statins	35 (21.1)	27 (23.1)	8 (16.3)	0.331
Diuretics	81 (48.8)	52 (44.4)	29 (59.2)	0.083
Antiplatelet therapy	25 (15.1)	16 (13.7)	9 (18.4)	0.441
ACE inhibitors/ARBs	80 (48.2)	51 (43.6)	29 (59.2)	0.067
TTR at 6 months of entry, n (%)	80 (60–100)	80 (60–100)	80 (60–83)	0.250
CHA_2_DS_2_-VASc score, median (IQR)	4 (3–5)	4 (3–5)	5 (4–6)	<0.001
HAS-BLED score, median (IQR)	2 (2–3)	2 (2–3)	3 (2–3)	<0.001

ACE inhibitors = angiotensin-converting-enzyme inhibitors; ARBs = angiotensin II receptor blockers; IQR = interquartile range; TIA = transient ischemic attack; TTR = time in therapeutic range.

**Table 2:** Crude risk of major adverse cardiovascular events (MACE) according to miRNAs.

	HR	95% CI	*p*-Value
miR-22-3p	1.07	1.02–1.14	0.013
miR-107	3.66	1.19–11.24	0.023
miR-146a-5p	0.86	0.74–0.99	0.042

HR = hazard ratio; CI = confidence interval.

**Table 3:** C-indexes, c-indexes comparison, integrated discrimination improvement (IDI), and net reclassification improvement (NRI) after the addition of miRNAs to the 2MACE score.

	C-index	95% CI	Z Score *	p *	IDI	95% CI	*p*	NRI	95% CI	*p*
2MACE	0.694	0.617–0.764								
+ miR-107 + miR-146a-5p	0.759	0.686–0.822	2.876	0.004	0.053	0.011/0.096	0.014	0.345	−0.327/0.518	0.736
+ miR-107 + miR-146a-5p+ miR-22-3p	0.762	0.689–0.825	2.518	0.012	0.056	0.012/0.101	0.015	0.047	−0.274/0.519	0.627

* for C-index comparison. CI = confidence interval; IDI = integrated discrimination improvement; NRI = net reclassification improvement.

## Supplementary Materials

The following are available online at https://www.mdpi.com/2077-0383/9/11/3645/s1, Table S1: Pilot study cohort baseline clinical characteristics.
