# Supplementary material for: Pilot Study on the Role of Circulating miRNAs for the Improvement of the Predictive Ability of the 2MACE Score in Patients with Atrial Fibrillation
Source: J Clin Med. 2020 Nov 12;9(11):3645. doi: 10.3390/jcm9113645 (PMC7698124; doi:10.3390/jcm9113645)
Supplement: Supplementary file 1 [file jcm-09-03645-s001.pdf]

Journal of Clinical Medicine

MDPI

**Supplementary Table 1.** Pilot study cohort baseline clinical characteristics.

| | Overall N = 19 | Controls N = 10 | Cases N = 9 | *p* |
|---|---|---|---|---|
| **Demographic** | | | | |
| Male sex, n (%) | 6 (31.6) | 4 (40.0) | 2 (22.2) | 0.405 |
| Age (years), median (IQR) | 80 (74-83) | 80 (74-84) | 80 (74-85) | 1.000 |
| **Comorbidities, n (%)** | | | | |
| Hypertension | 19 (100) | 10 (100) | 9 (100) | 1.000 |
| Diabetes mellitus | 4 (21.1) | 2 (20.0) | 2 (22.2) | 0.906 |
| Heart failure | 6 (31.6) | 3 (30.0) | 3 (33.3) | 0.876 |
| History of stroke/TIA/thromboembolism | 5 (26.3) | 3 (30.0) | 2 (22.2) | 0.701 |
| Renal impairment | 2 (10.5) | 2 (20.0) | 0 (0.0) | 0.156 |
| Coronary artery disease | 1 (5.3) | 0 (0.0) | 1 (11.1) | 0.279 |
| Hypercholesterolemia | 1 (5.3) | 1 (10.0) | 0 (0.0) | 0.330 |
| Current smoking habit | 1 (5.3) | 1 (10.0) | 0 (0.0) | 0.330 |
| Current alcohol consumption | 1 (5.3) | 1 (10.0) | 0 (0.0) | 0.330 |
| History of previous bleeding | 1 (5.3) | 0 (0.0) | 1 (11.1) | 0.279 |
| **Concomitant treatment, n (%)** | | | | |
| Amiodarone | 1 (5.3) | 0 (0.0) | 1 (11.1) | 0.279 |
| Digoxin | 4 (21.1) | 2 (20.0) | 2 (22.2) | 0.906 |
| Calcium antagonist | 1 (5.3) | 1 (10.0) | 0 (0.0) | 0.330 |
| Beta-blockers | 8 (42.1) | 3 (30.0) | 5 (55.6) | 0.260 |
| Statins | 2 (10.5) | 1 (10.0) | 1 (11.1) | 0.937 |
| Diuretics | 10 (52.6) | 2 (20.0) | 8 (88.9) | 0.003 |
| Antiplatelet therapy | 3 (15.8) | 2 (20.0) | 1 (11.1) | 0.596 |
| ACE inhibitors / ARBs | 11 (57.9) | 4 (40.0) | 7 (77.8) | 0.096 |
| TTR at 6 months of entry, n (%) | 83 (66-100) | 83 (78-100) | 75 (56-100) | 0.549 |
| $CHA_2DS_2$-VASc score, median (IQR) | 5.0 (3.0-6.0) | 4.5 (3.0-6.0) | 5.0 (3.5-5.5) | 0.968 |
| HAS-BLED score, median (IQR) | 2.0 (2.0-3.0) | 2.5 (2.0-4.0) | 2.0 (2.0-3.0) | 0.720 |

ACE inhibitors = angiotensin-converting-enzyme inhibitors; ARBs = angiotensin II receptor blockers; IQR = interquartile range; TIA = transient ischemic attack; TTR = time in therapeutic range.

© 2020 by the authors. Submitted for possible open access publication under the terms and conditions of the Creative Commons Attribution (CC BY) license (http://creativecommons.org/licenses/by/4.0/).
